# “I don’t belong here”: experiences of substance use and treatment compliance among young adults in Lango, Uganda

**DOI:** 10.3389/fpubh.2025.1677932

**Published:** 2025-10-22

**Authors:** Prossy Lynda Enon, Joan Nalunkuuma, Samuel Ouma

**Affiliations:** Department of Mental Health and Community Psychology, School of Psychology, Makerere University, Kampala, Uganda

**Keywords:** substance use disorders, health seeking, behaviors, treatment compliance, qualitative experiences, Uganda, young adults

## Abstract

**Introduction:**

Substance Use Disorders (SUDs) are a leading cause of disability and premature mortality among youth globally. A significant number of young people in sub-Saharan Africa (SSA) are reported to have SUDs and very few seeking help. The actual reasons for poor health-seeking behaviors and non-compliance remain largely unknown. The aim of this study was to examine the experiences of substance use, health seeking behaviors and treatment compliance among young adults diagnosed with SUDs in the Lango sub region of Uganda.

**Methods:**

This cross-sectional-qualitative study employed a phenomenological research design. Individual face-to-face audio-recorded Semi-structured interviews were conducted and thematically analyzed.

**Results:**

The study involved 10 participants aged between 18 and 35 years, four of whom were female. Key findings include participants’ early exposure to alcohol at home and through peer influence; involuntary health seeking behavior enforced by family and largely negative attitudes to SUD treatment under three major themes: 1) exposure to and maintenance of the use of substances; 2) circumstances of seeking treatment for SUDs; and 3) general ambivalence and negative attitudes toward treatment for SUDs.

**Discussion:**

Limited understanding of SUDs as serious health problems amidst increasing accessibility to more psychoactive substances in the community continues to impede health-seeking behavior and compliance with treatment. Concerted efforts aimed at increasing public mental health awareness of SUDs and innovative culturally sensitive clinical interventions can help reduce the morbidity and mortality associated with substance use.

## Introduction

The number of people using substances globally continues to increase and is projected to rise by 40% in sub-Saharan Africa (SSA) by 2030 (1). Young adults in particular face increased risks of use of various substances (2). In Uganda, for example, whereas peer networks that disapprove drinking may be protective, the intention to drink remains high among Ugandan youth (3). This may have far-reaching repercussions for the mental health of affected individuals, their families and the socioeconomic growth of broader communities as the demand for treatment is likely to increase. A recent review paper reported a disproportionately small number of people with SUDs in Africa received treatment (4). Moreover, available interventions for SUDs in SSA including prevention programs and their effectiveness for this growing vulnerable population have neither been widely tested nor extensively documented (5). Whereas the treatment environment and individual characteristics have previously been cited as predictive factors for recovery from SUDs in Uganda (6), the lived experiences of the affected people, especially youth, regarding those individual characteristics and other facilitating factors remain largely unexplored.

Substances including alcohol are widely consumed and only raise major concerns among the public when the user suffers serious behavioral and functional problems (7). It is likely that widespread social acceptance of consumption of alcohol in Uganda (2, 7, 8) might not only increase the likelihood of alcohol use disorder (AUD) but also expose, especially at-risk youth, to the use of other substances. This would result in more complex treatment needs and outcomes for affected individuals. Men with AUD for example, have previously been reported not to seek treatment, and when they do, to prefer other nonprofessional therapeutic means from spiritualists/traditional healers (9). Research on the treatment of SUDs in Uganda has not been extensively conducted despite increasing trends in the use of various substances, especially among young people (2). Successful treatment of SUDs may require both drastic and lifelong behavior changes (10) characterized by adherence and compliance with recommended treatment modalities. We are not aware of any study that has documented the experiences of treatment compliance for SUDs in Uganda. The absence of extensive public mental health research may affect efforts to find appropriate interventions for the growing number of people who might be struggling with SUDs.

Theoretically, the health belief model (HBM) advances concepts underpinning why persons suffering from conditions such as SUDs may not seek screening and treatment even when those services are available (11, 12). According to the HBM, the likelihood of behavior change is influenced by the cost–benefit analysis, wherein the individual weighs the expected benefits against perceived barriers (12). For many people with SUDs, the apparent barriers to behavior change may supersede the benefits. Consequently, continued misuse of substances presents several challenges with lifelong consequences, including a growing disabled, problematic and unproductive population. We therefore sought to explore the experiences of substance use, health-seeking behavior, and treatment compliance among youths receiving treatment for SUDs in the Lango subregion of Uganda.

## Materials and methods

### Study setting

Participants were recruited from Lira Regional Referral Hospital, a public facility serving mainly people from the eight districts of the Lango subregion. Anecdotal information from hospital administrators showed that the government-funded facility offers a wide range of general and specialized health care to an estimated 1,000 patients a day. Additionally, the psychiatric unit where participants were recruited was reported to have a daily attendance of more than 30 children and adults receiving various forms of mental health care.

### Study design, participants, and process

For this cross-sectional qualitative study, we adopted a phenomenological design. Specifically, interpretative phenomenological analysis (IPA) was adopted to explore the lived experiences of the participants. We sought to explore participants’ sense-making of experiences (13) of substance use and associated problems. The IPA approach has become especially useful for research examining experiences of health, patient illness and psychological distress in either cross-sectional (14) or longitudinal studies (15) and people’s interpretations of those experiences within their specific personal and social contexts (13). Given that exposure to, use of substances and the associated problems are events that have happened to our participants, we sought to examine the sense they have made of their experiences over the years, including treatment for SUDs. We argue that the meanings people with SUDs may attribute to their general experiences of substance use can act as barriers or facilitators to their treatment and compliance with the same, the phenomena examined in this study.

### Sample size and sampling strategy

Ten participants were purposively recruited between January and April 2023 from the mental health ward of a regional referral hospital by PLE. Participant inclusion criteria were as follows: aged between 18 and 35 years from the Lango subregion, on treatment for any SUD as the main diagnosis, mentally and physically stable, and willing to participate. Patients were excluded if they reported not currently receiving treatment for any SUD as the main diagnosis, were deemed not mentally and/or physically sound, or were not willing to take part in the interviews.

Although we anticipated interviewing up to 15 participants, saturation of key responses, such as reasons for admission to the hospital, issues surrounding hospitalization, problems associated with the use of substances (alcohol and/or cannabis), perceptions and attitudes toward treatment, and compliance with treatment for SUDs, was reached with the tenth participant. A decision to stop interviewing was reached by consensus between the interviewer (PLE) and the study supervisor (SO) when it became clear that the pattern and content of the responses had become similar, with no unique responses being elicited (16).

### Ethical considerations

This study was approved by the Institutional Review Committee of Gulu University (GUREC-2022-422) and received administrative approval from the study hospital prior to contacting participants. Potential participants were contacted while in the ward and given information about the study by the first author. Only those who consented in writing took part in the study. Privacy of participants was kept by conducting interviews in a private side room on the ward. Confidentiality was maintained by removing participant identifiers and limiting access to data on a password protected laptop to authors only.

### Data collection

Individual face-to-face audio-recorded Semi-structured interviews were conducted by PLE in either English or Leb Lango (a commonly spoken local language) with 10 purposively selected participants. Key sample questions on the semi-structured interview guide included “Tell me your experience with use of substance(s) before you sought help,” “What in your opinion made you seek help?” and “Tell me about your experience with the help you received from the hospital.” To ensure consistency in interviewing and minimize potential confounders arising from differences in interviewing, all 10 interviews were conducted by the first author, a native of the study area and fluent in both languages. Additionally, use of a semi-structured interview guide agreed to by the interviewer (PLE) and study supervisor (SO) prior to field work helped minimize bias during the recorded interviews. Audio recordings were subsequently transcribed verbatim by the first author, and those performed in the local language (7 interview transcripts) were translated into English before analysis. For purposes of accuracy, translated interviews were checked and validated by a language expert who was involved in the initial translation of study materials.

### Data analysis

Thematic analysis (TA) was performed manually by PLE and SO following Braun and Clark’s six-stage procedure of familiarization, coding, generating themes, reviewing themes, defining and naming themes and finally writing up the themes as findings (17). Specifically, employing a reflexive TA approach (18), we separately coded the data for initial meanings before embarking on an iterative, collaborative and interpretative reflexive process of generating themes. In this reflexive TA process, the theoretical influence (18) from both the IPA (13) and the HBM (12) is acknowledged. Additionally, we tapped into our subjective skills and interpretation of the participants’ experiences to actively and flexibly generate themes showing patterns of shared meaning of data, not our own consensus on meaning (19), as might be expected in coding reliability TAs and Codebook TAs (18). Our individual biases were nonetheless mitigated by ensuring that the emerging themes and subthemes were the outcome of reflexive thoughtful discussions (18, 19) supported by participants’ own reports and interpretation of their experiences through extensive use of excerpts from the interviews.

## Results

Table 1 shows 10 participants, four females and six males, aged between 18 and 35 years, participated in individual interviews exploring substance use, health-seeking behaviors and treatment compliance. The participants came from six of the eight districts of the Lango subregion in Uganda. The majority of the participants had attained at least secondary school education and used alcohol (9 participants) starting in childhood. Three participants reported using tobacco, while two used cannabis. The themes we present in the next section illustrate the participants’ experiences with exposure to and use of substances, problems characteristic of SUDs, and subsequent issues with treatment for those SUDs.

**Table 1:** Sociodemographic information and substances used.

Participant	Age	Gender	District	Education level	Marital status	Employment status	Substances used
A	18	Male	Lira	Secondary	Single	Unemployed	Alcohol, Tobacco and Cannabis
B	33	Male	Kole	Primary	Married	Peasant	Alcohol and Tobacco
C	31	Female	Apac	Tertiary	Single	Employed	Alcohol
D	35	Female	Dokolo	Secondary	Separated	Self Employed	Alcohol
E	28	Female	Oyam	Secondary	Single	Employed	Alcohol
F	27	Male	Kole	Secondary	Co habiting	Self Employed	Alcohol
G	23	Male	Oyam	Primary	Single	Employed	Tobacco and Cannabis
H	29	Male	Lira	Bachelor’s degree	Single	Unemployed	Alcohol
I	24	Female	Otuke	Secondary	Single	Unemployed	Alcohol
J	34	Male	Lira	Bachelor’s degree	Single	Unemployed	Alcohol and Tobacco

### Exposure to and maintenance of the use of substances

In this theme we highlight participants’ experiences of substance use. Participants reported early onset of substance use, starting in childhood. For some participants, the exact year when they first used substances, especially alcohol, was not clear. This was in part because of unusually early exposure within their homes, as we illustrate in the following subthemes.

#### Sociocultural factors

Given the availability and easy access to substances, especially alcohol, as both a socially acceptable drink and a source of livelihood for many families involved in brewing local alcohol, many children start to drink within their immediate and extended families.

*“I don’t remember, we just used to take wine from way back at home with my siblings and cousins. I cannot truly state that it started like this”.* (Participant E, 28-year-old female)

*“…I think I was around primary two or three. My mother brews and sells waragi* [local brew], *so we would also take some during the preparation. It is a normal thing at our home. Everyone consumes alcohol”.* (Participant B, 33-year-old male)

Alcohol use thus became normalized early in childhood as *“I used to take it just for leisure from way back when we were young”* (Participant D, 35-year-old female).

#### Peer pressure

For participants who were neither exposed to nor started to use substances at home, their already experienced peers played a significant role. Some of the participants reported that their first encounters were from peers at school or close communities as they socialized, often starting with less psychoactive substances and gradually taking more potent substances.

*“Well, we started with my friends in the village, we used to smoke pawpaws* [papaya leaves] *back then and later got access to weed from another friend of ours”.* (Participant G, 23-year-old male, used both tobacco and cannabis)

It seemed like the pressure to belong and fit in with peers meant that within quick succession, one was introduced to, and started to use multiple substances including alcohol, tobacco and cannabis.

*“Ah way back in high school, it was the thing, you know in boys’ school. Sometimes you just need to fit in”*. (Participant J, 34-year-old male university student)

*“…my friends who I drink with at the center smoke and they would give me to try, I don’t even know how I got used to it. My father also smokes tobacco rolls”*. (Participant B, 33-yearr-old male)

#### Perceived benefits

In addition to the pleasure initially sought from the use of substances, the participants reported several perceived benefits ranging from relieving stress to managing frustrations as well as an energy booster for those involved in manual jobs. For example, participant A then with a difficult home situation, early onset of use of multiple substances and current hospitalization following violent behavior noted “*used to use it* [Cannabis] *on the streets to pass the night”* following separation of his parents and “*ran away to look for her but could not… I was afraid to return home to my brutal father and his new wife*.” He emphasized, “*… I do not have a problem with it. It* [Cannabis] *actually saves me a lot.”* For some participants, the use of substances such as alcohol and tobacco was seen as both a source of energy and subsequently for relaxation or socializing after a hectic day’s work.

*“Well, here in the village, we use it for socializing. When you go to the trading center, you take some waragi and cigarettes with friends, and life moves on. You know garden work is hectic so we rest with some waragi”.* (Participant B, 33-year-old male farmer)

The above perceived benefits notwithstanding, the participants reported excessive use of substances as stressors and frustrations increased and their ability to cope waned.

*“It just increased four years ago when I separated with my husband. It has been one frustration after another since then. I have to fend for the children, and their alcoholic father does not even bother to check if they are eating, sick, or dead. So it* [alcohol] *has basically been my only source of comfort”.* (Participant D, 35-year-old separated female, with children)

### Circumstances of seeking treatment for SUDs

This theme highlights the health seeking behaviors and the circumstances that led to treatment for SUDs. The participants generally lacked insight into the consequences of the continued use of substances. Whereas their deteriorating health raised concerns among family members, many participants remained adamant about the need for health care. Occasionally, participants sought health care for what they considered to be serious medical symptoms. Some were brought to the hospital because of deteriorating health situations, including health emergencies resulting from intoxication, while others were involved in highly risky behaviors, as highlighted in the following subthemes.

#### Health scares due to intoxication with substances

A few participants voluntarily sought medical treatment for various complaints, such as headaches, nausea and bodily weakness, but not for SUDs. These participants, similar to others who were brought to the hospital unconscious, were also referred to the mental health unit following initial examinations for medical concerns. For example, participant D, a female participant, noted, “*I had not* [not sought help for alcohol abuse] *…it did not seem like a problem then. Am just realizing it’s a problem now.”* Her problem became apparent only when she was diagnosed with progressive liver damage. Similarly, participant C –a 31-year old female, also came to the hospital because *“had a very bad hangover, which did not treat me well. Therefore, it made me go to the hospital. At first, I thought it was malaria, so I went to the hospital.”* On assessment, she was suspected of having AUD and was thus referred to the mental health unit for further management.

Alcohol intoxication resulting in blackouts was commonly reported by the participants, leading them to be brought to the hospital for emergency medical attention. Once the initial health scare was stabilized and it became clear to the attending health workers that the blackout was substance induced, a referral for psychiatric evaluation was made.

*“I used to work in my uncle’s bar before he sacked me…I was going back home in the morning I think I was very drunk and I fainted on the road side…my mother called him and they brought me here”.* (Participant I, 24-year-old unemployed female)

*“I had increased my alcohol consumption and had kind of resumed smoking* [tobacco] *so that I could catch sleep after a hectic day. You know medical school is truly stressful, and sometimes you need to loosen up a bit. Therefore, it is either that I got so stressed and blacked out or the alcohol. I am not even sure at this point, but I just found myself at the hospital the other day”.* (Participant J, 34-year-old male medical student with both alcohol and tobacco use problems)

#### Involuntary hospitalization by concerned family members

Family and community members forcefully brought some participants to the mental health facility because of serious health concerns and involvement in risky behaviors. Additionally, the uncontrolled use of substances, domestic violence, and involvement in criminal acts prompted family members to seek help for their loved ones.

*“… I was just brought by my uncle who I worked for… He actually wanted to take me to prison that I stole and sold his goats but somehow brought me here claiming weed* [Cannabis] *had made me mad… The village people stoned and beat me calling me a mad person and a thief. They could have killed me if I had tried to resist being brought to the hospital”.* (Participant G, a 23-year-old male primary school dropout).

*“We just had a fight with my wife, and I decided to leave home to stay in our garden there in peace. My brothers and some village youths just came beat me badly and tied me, and I was taken to the hospital on a motorcycle even when I explained I was okay. I was beaten up and told if I don’t go to the hospital I would be imprisoned”.* (Participant B, 33-year-old male farmer)

We therefore observe that no participant voluntarily sought health care for SUDs. The poor health-seeking behavior could be attributed to several factors including lack of understanding of the dangers associated with substance use, denial, false hope and powerlessness over perceived causes such as witchcraft perceived by some participants as the cause of their troubles. For example, some participants, despite showing symptoms of SUDs, blamed family members for their health situation. “*It is what my step mother had planned already, to render me mad so that my father stops paying my school fees.”* (Participant A, 18-year-old male using alcohol, tobacco and cannabis). Others either underestimated or philosophized their situations.

*“I am just a drinker. It is not like I drink, and I mess myself like those other people who are even taken to* (name of hospital) *for drinking”.* (Participant F, 27-year-old male living with a partner)

*“…everybody knows, surprisingly these alcoholics know the effects of taking a lot of alcohol and for us smokers* [tobacco] *we also know about the lung cancer and stuff… but trust me, at some point we just use anyway hoping we won’t reach that stage and maybe by that time we will have stopped”.* (Participant J, medical student)

### General ambivalence and negative attitudes toward treatment for SUDs

This final theme shows the participants’ experiences of treatment for SUDs. The general ambivalence and negative attitudes toward health seeking affected the participants’ overall hospital admission experience and compliance with treatment for the SUDs, with most of them completely resenting treatment. However, we start with some benefits from previous treatment, as reported by a few of the participants. Gains in physical and mental health and improvements in family relationships were reported following previous treatment for SUDs.

*“It was helpful, because for me I used to think drinking would solve my problems, it will make me forget my problems, but it seems it was just increasing my problems. So these days I drink very little and I only do so to pass time but those days I would drink to relieve my small stress”.* (Participant C, 31-year-old female, single with tertiary education)

*“Maybe it was a good thing, because by the time I left the hospital, I went back home and all was well with me and my wife and children, my wife no longer threatens to leave and my friends who had deserted me are also slowly starting to talk to me”.* (Participant B, 33-year-old male)

Such reports of positive changes following hospitalization inadvertently show participants’ own acknowledgement of the struggles they faced with the use of substances, especially alcohol, albeit retrospectively. Most of them either denied or lacked insight into having SUDs. Current treatment was therefore characterized by several challenges with serious implications for compliance. Challenges ranged from medication side effects, feelings of misplacement/time waste, confinement and loss of time for productive activities, stigma, fear of being harmed by other patients and a lack of agency and respect for patients’ opinions, which consequently affected their compliance.

#### Poor treatment compliance

The initial treatment involving the use of medications, perhaps for detoxification, was a great challenge for most of the participants. They reported that the medicine administered to them made them weak and ill. This made it difficult for them to comply with treatment, as participants indicated that given the chance, they would stop the medication. However, we did not explore the specific medications they received.

*“…as you can see, I am always drowsy and sleepy. I think the medicine is meant to make people sleepy. It makes me feel unwell most of the time. It’s a terrible feeling”*. (Participant E, 28-year-old female treated for AUD)

The participants also reported cravings for substances of choice. This further compromised their ability to comply with available treatment.

*“I truly need the drink…I may even die here jokingly if we delay without getting out”.* (participant I, 24-year-old female, treated for AUD)

*“I feel headache majorly, and I am still sweating a lot at night. I can barely sleep too unless they give me the injection”.* (participant J, used both alcohol and tobacco)

#### Misplaced, confined, and wasted time

In part, due to the involuntary nature of hospitalization, lack of insight into the severity of SUD, continued hospitalization and treatment were perceived as some form of confinement.

*“Am just misplaced, I think. However, hopefully, I will be discharged tomorrow. This is my third day here. Am even wondering what am doing here. my wounds are even getting dry now; I think you people should just discharge us so I can heal from home”.* (Participant E, a 28-year-old female, single and employed treated for AUD)

Consequently, the hospitalization and treatment that followed was seen as a waste of time, neither warranted nor important for their recovery. Treatment compliance was thus only possible through what they perceived as coercion and/or confinement.

*“To be honest, I know I take a lot of alcohol, but to think I need to be in the hospital like sick people more so in a mental hospital is extreme, just a waste of time and money”.* (Participant I, 24-year-old female)

*“…truly hard. Now, for a person like me who is used to waking up and hustling, this feels like a real waste of time and imprisonment. I could have used this time to do something more productive”.* (Participant F, 27-year-old self-employed male)

*“These people are just wasting my time. It is mad* [mentally ill] *people who are supposed to be treated here not me…, me am not mad, I can assure you”.* (Participant G, Tobacco and cannabis)

For participants who acknowledged having problems, the attribution of their conditions to external negative influences from jealous relatives and friends made them want to seek further help outside the hospital.

*“…I need to go home and get to the root of the things that happened to me. This is not ordinary and not even medical. Someone is fighting my career. There is no mad person in my lineage; how can I be the first one?”* (Participant F, 27-year-old self-employed male)

When asked if they would seek help from the hospital in the future for SUD or related mental illness, participants who had minimized the impact of SUDs on their well-being were still noncommittal and disregarded the need for health seeking, as illustrated in excerpts below.

*“…it depends, but I doubt I will ever get a problem that can lead me to want to come here because I don’t see my self-smoking* [tobacco] *to that point. This is going to be my last time here. In addition, if it was not for the force, I would not have ever come here”*. (Participant J, Alcohol and Tobacco)

*“If it brings me bigger problems even after limiting, I will go. If it does not bring a big problem, I will not go, but I will do exercises that chase away the hangover”.* (Participant C, 31-year-old employed female).

#### Perceived sigma as a key barrier to treatment

Stigma was another important factor that affected participants’ attitudes toward seeking and continuing care at mental health facilities. The participants reported ongoing stigma, including self-stigma, and stigma from family and community. About use of alcohol, one participant’s self-stigma was highlighted by “*I am embarrassed. She pays part of my fees and telling her I got drunk to a point of losing my mind. At this point I do not really want anyone coming around to judge me. I just want to be alone”* (Participant J, 34-year-old male). Asked about the challenges faced while receiving treatment for SUDs and how care could be improved, another participant reported:

“*I just be anxious all the time fearing my fans will see me. I really need to go* and “*first they need to transfer the unit to a really private place where not many people can see the patients. Otherwise leaving it here in the middle of the town, trust me no one can want to come here and be seen* respectively” (Participant F, 27-year-old male).

These excerpts point to the self-stigma that could have undermined the participants’ health seeking behaviors and compliance to the available treatment. Family stigma was also reported. Some participants reported being ostracized by their own families because of substance use.

*“In fact, it even became harder when I got home. Everyone started blaming me for dropping out of school and drinking a lot of alcohol…calling me lazy and irresponsible. I would just stay alone and not talk to anyone”.* (Participant H, 29-year-old male)

Because of perceived or actual community stigma, some participants did not want to be identified with the facility because they feared being labeled “mad” and losing respect.

*“For us anyone who we see here is mad. Now am sure my enemies are out there, spreading it to my fans. I truly need to leave before more people see me here; I am uncomfortable for real”.* (Participant F, 27-year-old self-employed male, in entertainment industry)

*“You know people think us who smoke we are bad people and that we are thieves and yet sometimes it is not us who do those bad things. Like me I just smoke for fun with my friends”.* (Participant G, 23-year-old male)

The participants’ reports above seemed like the general community negative attitudes about SUDs treatment. Their observations show possible widespread perceived community stigma about mental health care in general. Participants also reported poor reception from the health workers.

*“…to the service providers, they should also accept people like us who take alcohol. Because if the body is used to something or you are addicted, they should accept us when we go to the hospital. They should attend to us not rebuke us. For example, when you were advised to stop and you still come back with the same problem they usually just shout at you. So we urge them to handle us slowly until the drinking habit leaves us”.* (Participant C, 31-year-old female)

In keeping with their perceived forced hospitalization and overall stigma, the participants also reported not being listened to and lacking agency in their own treatment. According to them, the caretakers’ opinions were respected more than their own. These feelings and thoughts strengthened the participants’ resolve to resist treatment.

*“…first of all, I don’t belong here; second, they treat people like mad people who don’t know what they are saying, everything they do forcefully. Yesterday when I tried to leave and go back home, the askari* [security guard] *twisted my arms, up to now am still feeling the pain”.* (Participant A, 18-year-old male with multiple substance use).

In summary, we note early exposure to substances. Most of the participants lacked insight on problems associated with their use of substances compromising health seeking behaviors. Efforts to care were therefore characterized by an overarching theme of forceful interventions, especially for alcohol- and cannabis-related problems, right from admission to the hospital throughout the treatment process. Widespread experiences of stigma from self, family and community by both male and female participants could have contributed to the general ambivalence and negative attitudes participants had regarding SUD treatment and care. These experiences further characterized the low compliance with treatment observed among our participants.

## Discussion

In this qualitative study, we explored experiences of substance use right from onset through to the problems associated with substance use and seeking treatment for SUDs. The participants were young adults aged 35 and younger. The findings show experiences of use of alcohol, cannabis and tobacco resulting from early exposure within family settings and peer influence. Though largely lacking insight, both male and female participants reported various behavioral and functional problems characteristic of SUDs. Alcohol was the most commonly used substance with an onset of consumption in early childhood as a widely socially acceptable drink, similar to other parts of Uganda (2, 8, 20). By adolescence, several of our respondents were using alcohol in large quantities. Peer influence only exacerbated the risk of AUD as well as exposure to tobacco and cannabis, which were obtained mainly outside the participants’ homes. Unlike AUD, which presented various health concerns, cannabis was associated with violent behavior prior to involuntary hospitalization enforced by family and close community members. The violent behavior potentially reflecting psychosis could be attributed to the early onset of cannabis use (21) or likely heavy use of the substance (22). However, we did not assess participants during recruitment for comorbidities such as psychosis, only excluding those who showed obvious signs of being mentally unstable. Despite a small number of participants reporting the use of illicit cannabis, the risks of its early onset and heavy use could raise further concerns, especially for their communities. Additionally, the legal implications for our participants using cannabis, some of whom were threatened with imprisonment if they did not comply with involuntary hospitalization, remain unclear. Whether threats of possible incarceration and the circumstances leading to hospitalization influenced the experiences of the participants reported in our interviews cannot be fully ascertained.

The participants reported using substances, especially alcohol, for various reasons (8). The findings of the current study show that in addition to the lure of pleasure sought in the use of substances, especially during adolescence (23), young adults continued to use substances to cope with life stressors (8, 24), such as relaxation after hard work in crop fields. People with a history of early childhood-onset of use of substances such as alcohol and cannabis, amidst several perceived benefits, might thus find it hard to recognize the severity of SUDs that gradually worsened over the years. Seeking treatment for a condition that is neither perceived as a problem nor seen as having a serious impact on individual well-being would thus not come easy (7). It is therefore not surprising that the participants either sought medical attention following serious physical health issues or were involuntarily brought to the hospital by concerned family members. In both cases however, uncontrolled substance use was a key trigger unknown to most of the participants.

We also noted social factors associated with involuntary psychiatric hospitalization (25–27) among our respondents. Stigma was a key barrier to both health seeking and compliance with treatment among our participants. Ongoing experiences of actual or perceived stigma –self-stigma, from family and community including health workers, became strong barriers to sustainable and meaningful care of the participants. Family and community stigma in particular meant reduced perceived social support from significant others. For participants who were aggressively brought to the hospital, prospects of returning and continuing with recovery at home were slim due to perceived apparent poor social support. Suspected criminal behavior due to substance use at the time of hospitalization could also lead to recovering individuals not being received back home as patients in need of care but rather as community outcasts. Such community attitudes may have unintended outcomes. First, they would worsen feelings of loneliness among the already socially isolated individuals. Second, they would further compromise not only SUD recovery efforts but also the mental health of the affected young adults. Finally, negative community reception would complicate any future motivations and efforts to health seeking and treatment of SUDs.

Lango sub region in Uganda is witnessing an increase in substance use like many other parts in SSAe (2, 28, 29). The resulting risks of SUDs as observed among the young adults in this study require urgent and concerted efforts to reverse the negative trends. Unfortunately, health seeking efforts by families of the affected individuals are compromised when those presenting with SUDs lack insight into the severity of their health situations. In the affected neighborhoods, young adults with SUDs may inadvertently perceive their health situations as comparatively better than several others and thus think of themselves as not requiring help. This was the case with some of our participants who reported that admission to the mental hospital was a waste of time and as such did not think they belonged there.

Moreover, owing to the likely interaction of various social-ecological factors, such as poor economic situations and broader cultural beliefs, the participants lacked many of the elements of the health belief model necessary for health-seeking behavior (12). For example, at the individual level, the participants hardly perceived any threat from the continued use of substances due to their long-standing poor perceptions of their own susceptibility and severity of SUDs. This made many of them increasingly skeptical about having any SUD further compromising chances of seeking and/or accepting help at the mental health facility.

With respect to the perceived benefit of action argued in the HBM (12), the majority of the participants viewed hospitalization and treatment as useless and a waste of time. The perceived barriers to action such as our respondents’ lack of awareness of symptoms/cues of action, denial and stigma from self and the wider society (30) evidently compromised any possible perceived benefits of hospitalization. People diagnosed with AUD have been reported to perceive greater public stigma (31). We observed that involuntary health seeking by family members triggered by either serious physical health symptoms among participants or substance induced violent and bizarre behaviors, similar to findings by Belete et al. (32), could not foster positive attitudes toward health-seeking behavior in particular and SUDs treatment in general. Self-efficacy—a belief in one’s ability to successfully attain the desired behavior (11)—was not evident in most of our interviews with participants either. Consequently, participants not only felt misplaced at the hospital but also saw no need for the care they were subjected to during hospitalization.

Similar to the findings of Glowacki et al. (33), negative experiences characterized interactions between health workers and our participants. Our respondents viewed situations that caused them to visit the hospital as health emergencies and therefore, once stabilized, demanded immediate discharge. A typically prolonged stay in the hospital to address the underlying issues of SUD was thus unimaginable and very unlikely for many. This general ambivalence toward treatment derailed our participants’ much needed motivation for behavior change and recovery from SUDs (10). Compliance with available treatment could thus neither be achieved nor sustained beyond the hospital where the respondents were currently admitted. This put the participants’ mental health in jeopardy as the few gains made during their brief hospitalization could quickly be eroded upon return to their homes leading to relapses and re-hospitalization (34). Moreover, family members’ despair at inevitable relapses and related substance use behavioral or functional problems may diminish all possible efforts toward future treatment, resulting in more severe health and social ramifications for the affected young adults, their families and communities.

Measures aimed at raising awareness about the dangers of substance use and innovative interventions for already affected young people can yield promising results (34–37). To enhance services for people with SUDs, other complex associated problems must be equally screened and addressed (20, 38). The conventionally available medical detoxification and management of the physiological symptoms of intoxication offered to the participants, who generally lacked insight into the severity of their SUDs, seemed ineffective in increasing their uptake of treatment. The absence of interventions focusing on behavioral change and motivational interviewing thus continues to hinder the possible recovery of young adults with SUDs. Consequently, the increasing prevalence of substance use across the Lango subregion in particular and Uganda in general (2, 7, 8) portends precarious public mental health situations for a substantial proportion of Uganda’s young adults.

### Study limitations

We note a few limitations to our study. First, by focusing on a small sample from one subregion of Uganda, our findings are grounded in the population that sought help from mental health facilities and therefore may not be generalizable to the entire subregion or other geographical areas. Second, although we set out to explore the experiences of a wide range of substances, our participants reported issues mostly concerning the use of alcohol and cannabis. These experiences may therefore not represent other substance use disorders. Finally, we did not examine the nature of the treatment for substance use disorders the participants received. Overall, the absence of a specialized substance treatment facility with qualified multidisciplinary health staff could have contributed to the negative experiences of treatment for SUDs reported by our respondents.

## Conclusion

In this study, we have examined the experiences of onset of substance use as well as behavioral and functional problems associated with especially alcohol and cannabis use. Sociocultural factors and peer influence play significant roles in early exposure to, and maintenance of substance use among young people. Failure to recognize the severity of SUDs in part due to early exposure to substances and the meanings attached to the use of those substances foster poor health-seeking behaviors. The stigma associated with mental illnesses in general remains a significant barrier to seeking and complying with available interventions for SUDs in a general mental health facility. Consequently, negative attitudes toward care and low compliance with available treatment for SUDs are widespread.

### Recommendations and future directions

Public health campaigns aimed at increasing mental health awareness, specifically SUDs are urgently needed. Such campaigns should aim at demonstrating links between substance use and extant traditional cultural practices as well as promoting positive ways of coping with prevailing life challenges rather than resorting to the use of substances. Additionally, innovative mental health clinical interventions involving peer support and culturally sensitive practices cane helpcurb the increasing risks of morbidity and mortality among young people in the affected communities of the Lango sub region in Uganda.

## Data Availability

The datasets presented in this study can be found in online repositories. The names of the repository/repositories and accession number(s) can be found at: https://data.mendeley.com/datasets/dxbxzfkx98/1.
